# Application of high-resolution mass spectrometry to determination of baclofen in a case of fatal intoxication

**DOI:** 10.1007/s11419-016-0314-0

**Published:** 2016-03-29

**Authors:** Paweł Szpot, Agnieszka Chłopaś, Grzegorz Buszewicz, Grzegorz Teresiński

**Affiliations:** Department of Forensic Medicine, Wroclaw Medical University, ul. J. Mikulicza-Radeckiego 4, 50-345 Wrocław, Poland; Department of Forensic Medicine, Medical University of Lublin, ul. Jaczewskiego 8b, 20-090 Lublin, Poland

**Keywords:** Baclofen, Fatal intoxication, QTOF, LC–MS, HRMS

## Abstract

The study focused on the application of high-resolution mass spectrometry (HRMS) to postmortem toxicological analysis. Fast and simple sample preparation involved precipitation with acetonitrile, removal of phospholipids using special columns and filtration. Qualitative and quantitative analyses were performed using ultra-performance liquid chromatography coupled with quadrupole time-of–flight mass spectrometry. The method was validated by determining the limit of quantification, precision, recovery and matrix effect. The use of a high-resolution spectrometer allowed us to determine the precise masses of the fragments of interest and to suggest the fragmentation pathway of baclofen. The usefulness, effectiveness and assets of the procedure were confirmed by an authentic case of a 25-year-old woman fatally intoxicated with baclofen who was found dead in her apartment. Toxicological analysis of postmortem blood samples demonstrated that the baclofen concentration was 30.7 μg/mL. In only one published case describing fatal baclofen intoxication were no other xenobiotics (that could interact with baclofen) found. To our knowledge, this is the first report dealing with analysis of baclofen by HRMS.

## Introduction

**Fig. 1:**
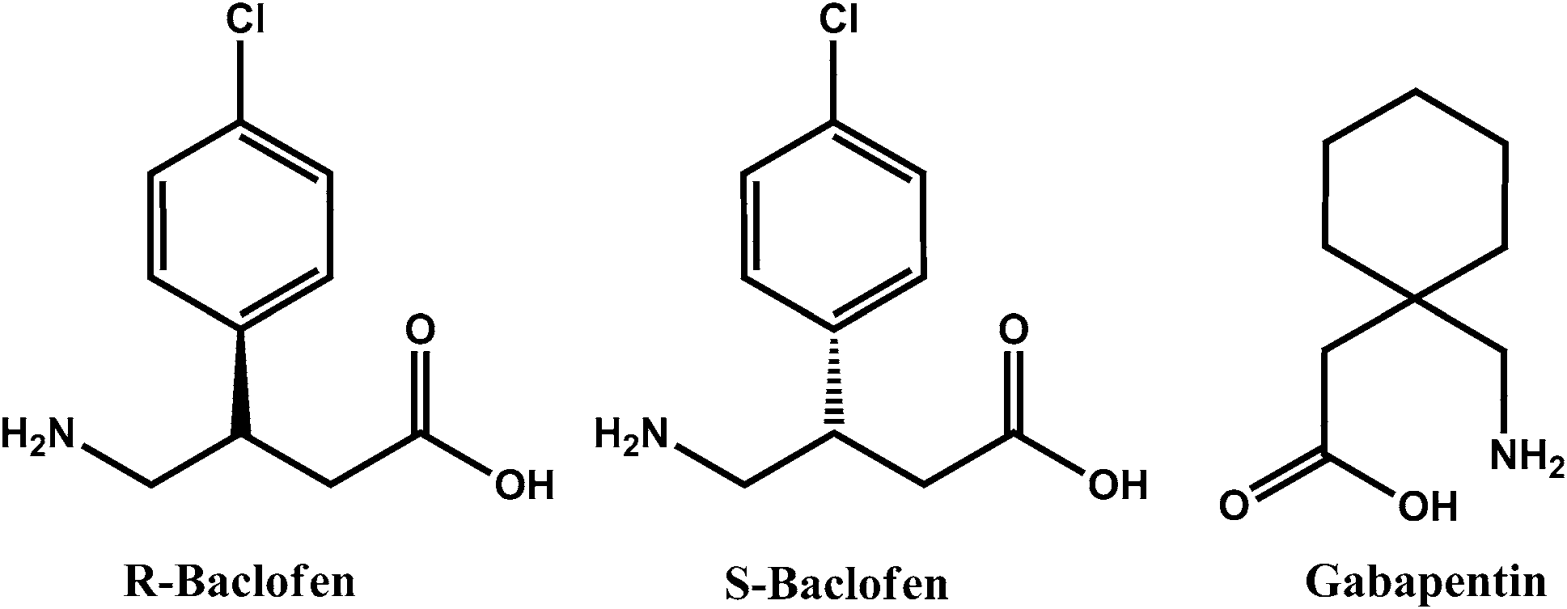
Structures of enantiomers of baclofen and the internal standard (IS) gabapentin

Baclofen [(±)-4-amino-3-(4-chlorophenyl) butanoic acid, β-(aminomethyl)-4-chlorobenzenpropane acid, Fig. 1], with commercial names of Atrofen^*®*^, Baclofen^*®*^, Lioresal^*®*^ and Lyflex^*®*^. Baclofen is a drug affecting the central nervous system (CNS). The mechanism of action of baclofen, which is a derivative of γ-aminobutyric acid (GABA), involves stimulation of GABA_B_-ergic receptors located pre- and postsynaptically [1]. The substance decreases the skeletal muscle tone by inhibiting the mono- and polysynaptic reflexes at the spinal cord level, but does not affect the neuromuscular conduction [2]. Baclofen is used for the treatment of spinal cord diseases, cerebral stroke and cerebrospinal meningitis. It is also applied in patients with severe chronic spasticity in multiple sclerosis [3, 4]. Moreover, the drug is known to minimize the symptoms of alcohol craving [5–7].

Baclofen occurs as a mixture of two biologically active enantiomers (R and S), that differ in the arrangement of substituents around a chiral carbon atom. It has been found that the R(−) isomer of baclofen exhibits about 100-fold more potent pharmacological action than its S(+) form [8]. Wuis et al. [9] compared the pharmacokinetics of racemic baclofen and its two enantiomers, and they have found that the biological half-life (*t*½) of R(−) baclofen is 4.5 h, which is longer than the half-life of its racemic mixture (3.8 h) as well as S–baclofen (2.9 h). These findings suggest the existence of stereoselective renal elimination pathways [9]. After its oral administration, baclofen is rapidly and completely absorbed. It reaches the maximum blood concentration after 2–3 h. The biological half-life is 2.5–4 h [10]. It is bound to blood proteins at a rate of about 30 %. Due to deamination and oxidation, 15 % of the absorbed dose of baclofen is metabolized in the liver to β-(*p*-chlorophenyl)-γ-hydroxybutyric acid whereas 85 % is excreted intact into urine [11]. The oral therapeutic dose for adults should be tailored individually and ranges between 15–80 mg/day [3]; significant complications, and life-threatening cases have been reported with doses as low as 300 mg [12].

The literature reports of acute and fatal baclofen intoxication are rare, as compared to other CNS-affecting drugs [13, 14]. In some cases, the symptoms of overdose occur already during the use of the therapeutic dose and include decreases in muscle tone, dizziness, sedation, seizures, loss of consciousness, hypothermia, inhibition of the respiratory function, apnoea and coma [15]. Long-term administration followed by withdrawal can lead to psychotic, manic and paranoid anxiety disorders [16].

To date, baclofen has been determined in various biological materials using the techniques based on gas [17, 18] or liquid [19–21] chromatography, liquid chromatography–tandem mass spectrometry [22–25] as well as capillary electrophoresis [26]. However, the majority of these methods require time-consuming sample preparation. As far as medico-legal toxicology is concerned, it is essential to design a simple and fast analytical procedure, providing reliable results.

Substantial advances in the development of special devices for identification and determination of chemical compounds in biological material have recently been observed. Thanks to equipment, increasingly more substances at markedly lower concentrations can be detected. However, considering the interpretation of results and established norms regarding, e.g. validation of methods, much more attention should be paid to toxicological analysis; therefore, numerous recent descriptions contain detailed data regarding analytical problems associated with examinations of the postmortem material [27–29]. In this manuscript, high-resolutions mass spectrometry (HRMS) was used for the determination of baclofen in postmortem blood. The technique based on liquid chromatography–hybrid quadrupole time-of-flight-mass spectrometry (LC–QTOF-MS) enables comprehensive targeted forensic screening. The use of this detector allows one to determine the exact weight and elemental composition of an unknown compound. To our knowledge, this is the first paper describing the determination of baclofen in postmortem blood by LC–QTOF-MS. The aim of the study was to develop and validate a highly specific, simple and quick method of determination of baclofen in biological material, to analyze fragmentation of this substance and to review the literature concerning baclofen intoxication cases.

## Materials and methods

### Chemicals and reagents

Water (Chromasolv^®^ LC–MS), acetonitrile (Chromasolv^®^ LC–MS), methanol (Chromasolv^®^ LC–MS), gabapentin and formic acid were purchased from Sigma-Aldrich (Steinheim, Germany); (±)–baclofen was purchased from Polpharma SA (Starogard Gdański, Poland). Standard solutions of baclofen were prepared in water. Gabapentin [internal standard(IS)] was prepared in methanol. The standard solutions were stored in a refrigerator at −20 °C.

### Blank human blood material

Four blank samples of postmortem human blood were derived from the Chair and Department of Forensic Medicine. Blank samples did not contain any anticoagulant and were screened prior to spiking to ensure that they were free from baclofen and gabapentin.

### Chromatographic conditions

Chromatographic analysis was performed using an ultra-high performance liquid chromatograph (UHPLC Infinity 1290, Agilent Technologies, Waldbronn, Germany). The separation was done employing a Poroshell 120 EC-C18 column (3.0 × 100 mm); 2.7 μm (Agilent Technologies, Santa Clara, NM, USA).

A mixture of 0.1 % formic acid in water (A) and 0.1 % formic acid in acetonitrile (B) was used as a mobile phase. The gradient elution was carried out at constant flow rate of 0.4 mL/min in the following gradient mode: 0 min (95 % A/5 % B); 0.5 min (70 % A/30 % B), 5 min (30 % A/70 % B), 7 min (5 % A/95 % B) and 8 min (95 % A/5 % B). Return to the initial conditions (95 %A) was achieved 2.5 min after the 8-min long analysis. The injection volume was 5 μL.

### Mass spectrometry

Detection of the study compounds was achieved using a quadrupole time-of-flight (QTOF) mass spectrometer (QTOF 6540, Agilent Technologies, Santa Clara, NM, USA). The spectrometer was equipped with an electrospray ionization (ESI) source. The source conditions were as follows: gas temperature 350 °C; gas flow 10 L/min.; nebulizer (N_2_) 35 psi; sheath gas temperature 400 °C; sheath gas flow 12 L/min.; capillary voltage 3000 V; fragmentor voltage 140 V; skimmer voltage 65 V; oct 1 RF Vpp 750 V; mass range *m*/*z* 50–1000. Quantitative and qualitative analyses were carried out in the MS mode. To ensure accuracy of mass measurements, reference mass correction was used. Masses at *m*/*z* 121.0509 and 922.0098 were used as reference ions. The mass tolerance used for the extracted ion chromatograms was ±20 ppm. The mass error for the reference masses used prior to analysis was lower than 2.7 ppm.

The analysis of product ion formation of baclofen at a concentration of 5 μg/mL was carried out in the tandem QTOF mode with spectral parameters: mass range *m*/*z* 50–1000; acquisition rate 1.5 spectra/s; isolation width *m*/*z* ~4; and collision energies (CEs) 5, 20 and 35 V. Retention times for baclofen and gabapentin were 2.02 and 1.96 min, respectively. Positive ionization was performed. Quantitative and qualitative ions for determinations of baclofen and gabapentin were *m*/*z* 214.0629, 151.0309 and *m*/*z* 172.1332, 154.1226, respectively.

### Sample preparation

A 200-μL volume of postmortem blood was poured into a 2-mL Ependorff tube; then 20 μL of gabapentin (IS) at a concentration of 10 μg/mL was added and mixed. The blood prepared in such a way was precipitated by adding drops of 400 μL of frozen 0.1 % formic acid in acetonitrile (constantly mixed) and the mixing was continued for 30 s. The samples were centrifuged for 15 min at 20,627*g* at 5 °C (2–16 K, Sigma, Osterode am Harz, Germany). Subsequently, 400 μL of the supernatant was loaded onto a column of Phree Phospholipid Removal 1 mL (Phenomenex, Torrance, CA, USA) coupled with a polytetrafluoroethylene syringe filter (pore size 0.22 µm, FilterBio, Nantong, China) and filtered using a Visiprep vacuum manifold (Supelco, Bellefonte, PA, USA) under pressure of 10 mmHg. A 100-μL volume of each of the extracts was transferred to the inserts and analyzed using LC–QTOF-MS.

### Method validation

Method validation was carried out following the EURACHEM guidelines [30]. The method was validated by determining the following parameters: specificity, precision, recovery, limit of quantification (LOQ) and matrix effect. The postmortem blood was chosen as a target matrix for method validation. The specificity of the method was evaluated by analyzing a blank sample and a blank sample spiked with the LOQ levels of baclofen and IS. Calibration linearity was verified by analyzing each of four different blank blood samples with the additions of baclofen at the concentrations of 1, 2, 5, 10, 20 and 50 µg/mL. Precision was expressed using % relative standard deviation (%RSD) for each calibration curve point analyzed in four repeats. Recovery and the matrix effect were determined for four points of the calibration curve: 1, 5, 10 and 20 µg/mL. Recovery was determined by adding 20 µL of baclofen and IS, and 400 µL of frozen 0.1 % formic acid in acetonitrile to 180 µL of water. The solution of standards prepared in this way was analyzed using LC–QTOF-MS, and the data were compared with each of the four calibration curves in postmortem blood. The LOQ in this method was the blood concentration of baclofen for which both quantitative and qualitative ions could be observed and the standard deviation (SD) of the concentration did not exceed 20 %. To calculate the matrix effect, the formula by Chambers et al. [31] was applied:

$$ {\text{\%  Matrix effect }} = \left( {\frac{\text{Response extracted sample}}{\text{Response standard}} - 1} \right) \times \;100. $$

The negative result evidences suppression while the positive result evidences enhancement of analyte signals. The validated method was used for analysis of blood samples sent to the Chair and Department of Forensic Medicine to determine the cause of death.

## Case history

A 25-year-old woman was found dead in her apartment. On the day of death, the woman consumed an undetermined amount of drug(s) of unknown origin, which most likely caused her death. Near the deceased, the following containers and baclofen tablets were found: one white tablet, 5 mm in diameter, one empty Baclofen 25 mg container for 50 tablets, one full container of the same drug with 51 tablets and one container with 12 and 1/2 tablets. The above suggested that the woman took 87 and 1/2 tablets (maximal potential dose 2187.5 mg). The case investigation revealed that the woman abused alcohol, often had arguments and caused various problems. Before her death, she wrote to her boyfriend informing him that she took 100 tablets of baclofen. During the prosecutor’s investigation it was established that multiple times after arguments with her boyfriend, the woman had taken various drugs, had drunk alcohol and subsequently vomited.

External examination and autopsy did not reveal any pathological changes. There was no ethyl alcohol present in her blood. Autopsy results excluded death due to mechanical trauma, acute alcohol intoxication and diseases.

In this case, postmortem venous blood samples from the femoral vein were collected via a chemically pure plastic tube, imblood samples indicated that there were impure compoundsmediately frozen at −22 °C, and stored until analysis. No anticoagulants were added to the sample.

## Results and discussion

**Fig. 2:**
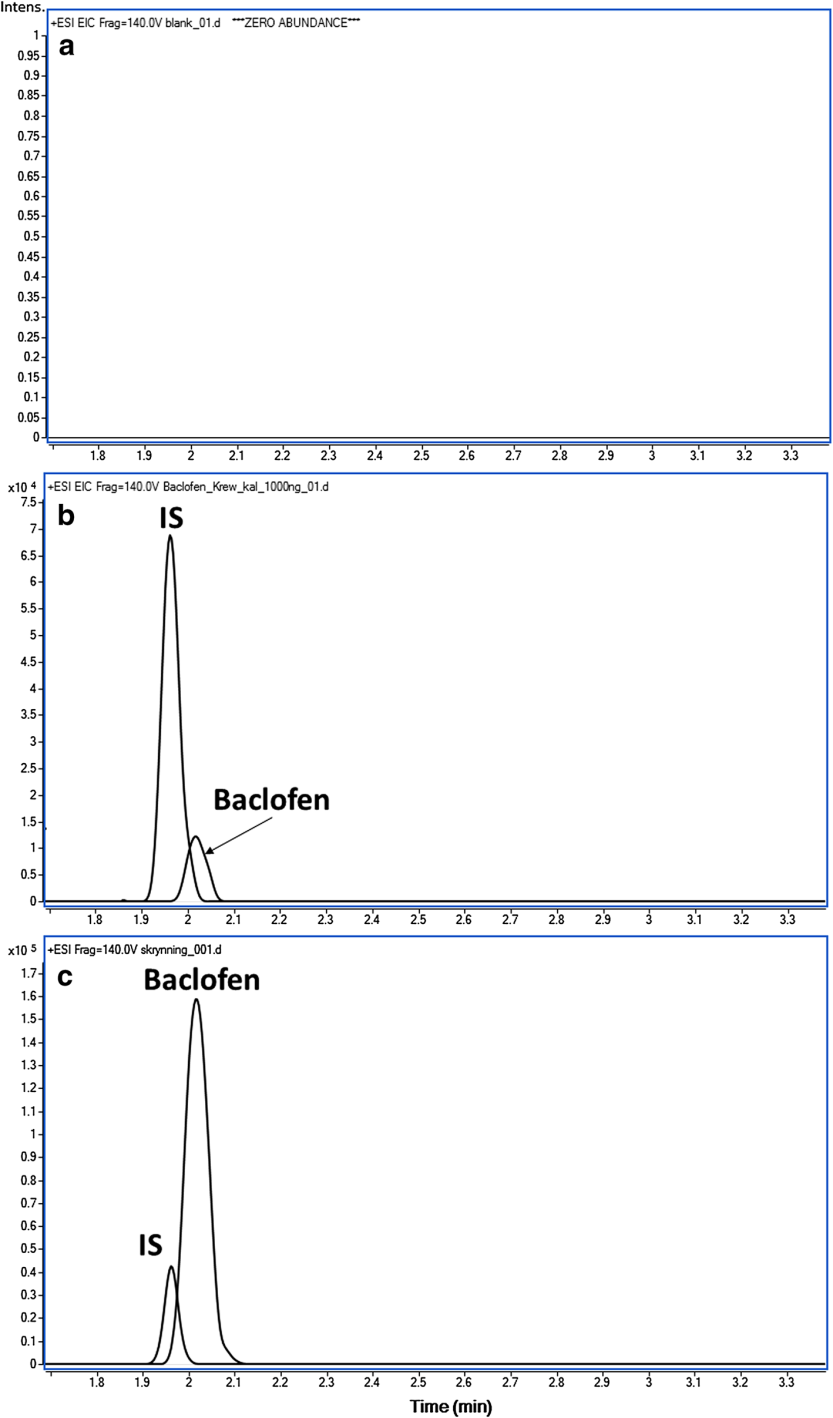
High-resolution extracted ion chromatograms obtained by liquid chromatography–quadrupole time-of-flight-mass spectrometry for a blank blood sample (**a**), blank blood sample spiked with 1.0-µg/mL baclofen and 1.0-µg/mL IS (**b**), and the authentic postmortem blood sample in the present case spiked with 1.0-µg/mL IS (**c**) using the precursor ions of baclofen and IS at a collision energy (CE) of 5 V

The use of HRMS confers high specificity and reduces the risk of potential interferences related to the complexity of the matrix. The absence of chromatographic peaks at the same retention times as those of target substances in blank blood samples (Fig. 2a) indicated that there were no impure compounds that might give false positive signals in these blank samples. The LOQ for the method was 1 µg/mL, whereas the coefficient of determination (*R*^2^) for the calibration curve was 0.996. Table 1 presents concentrations, SDs and precisions for individual calibration curve points. %RSD for the method was 5.1–15.3 %.

**Table 1:** Calculated concentrations in the calibration curve for baclofen in postmortem blood

Nominal concentration (µg/mL)	Level	Calculated concentration (µg/mL)^a^	SD	Precision (%RSD)
1	1	0.91	0.14	15.3
2	2	2.12	0.20	9.82
5	3	4.99	0.54	10.8
10	4	9.97	0.51	5.08
20	5	20.0	1.21	6.06
50	6	50.4	4.67	9.27

*SD* standard deviation, *RSD* relative standard deviation

^a^Mean of four repeats

Table 2 shows the mean percent recovery rates and mean matrix effect at four different concentrations of baclofen in postmortem blood.

**Table 2:** Recovery rates and matrix effect of baclofen in postmortem blood

Nominal concentration (µg/mL)	Recovery rate (%)^a^	Matrix effect rate (%)^a^
1	104	−40.9
5	90.3	−50.5
10	97.3	−50.3
20	109	−38.2

^a^Mean of four repeats

The method described enabled detection of baclofen at a level lower than the LOQ, i.e. 1 µg/mL. The quantitative and qualitative ions were also observed at the concentration of 0.5 µg/mL; however, at such a wide range of concentrations (0.5–50 µg/mL), the concentration calculation error for this point was over 20 %; moreover, reduced linearity was observed. Therefore, for concentrations up to 0.5 µg/mL, a separate calibration curve should be constructed. It can be assumed that the concentration at 0.5 µg/mL of baclofen in blood was a limit of detection (LOD). The recovery rates in samples were 90.3–109 %, which confirms that the use of gabapentin as an IS was the right decision (used earlier for determination of baclofen in rat materials by Kim et al. [32] with recoveries of 93.2–100.4 %). The negative matrix effects evidenced suppression of analyte signals, which could not be completely removed despite the use of precipitation, sorbent-removing proteins and phospholipids as well as filtration during sample preparation.

The presented method has a limitation especially for quantification of baclofen in postmortem blood. Gabapentin is clinically used for treating epilepsy; as such, there is potential influence from originally occurring gabapentin. For this reason, it is important to first analyze the sample without the IS–gabapentin when using this method.

Qualitative analysis was based on the automatic comparison of all ions from the total ion chromatogram to our database. Crucial criteria included retention time (±0.5 min.) and mass error (±2 ppm). The qualitative analysis showed a positive result for baclofen, which was confirmed by comparison of examined blood samples with the reference standard of baclofen by product ion mass spectra, with parameters of the precursor ion at *m*/*z* 214.0629, fragmentor voltage at 140 V and CE at 20 V. The concentration of baclofen determined in the postmortem blood was 30.7 µg/mL (*n* = 4, SD of 1.8 μg/mL; %RSD of 5.94; Fig. 2c). Tested blood was screened prior to analysis in order to ensure that it was free from gabapentin. Screening testing did not indicate other compounds in the biological material.

**Fig. 3:**
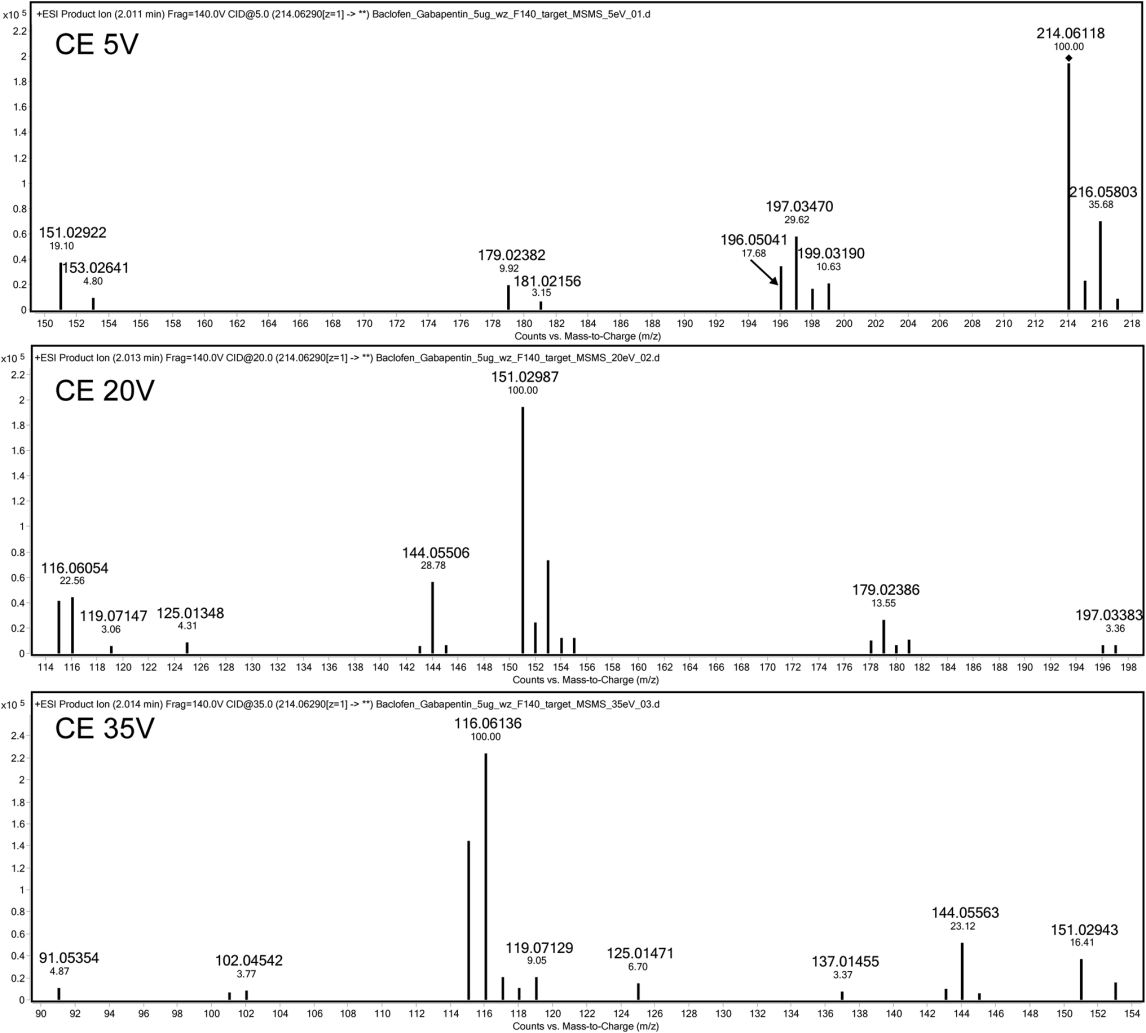
High-resolution product ion mass spectra of baclofen obtained at different CEs. The accurate mass of the precursor protonated baclofen was 214.0629

The application of a QTOF detector enabled obtaining high-resolution mass spectra (MS/MS). The MS/MS analysis allowed us to investigate the mechanism of product ion formation from baclofen, which, to our knowledge, has not been described before. The obtained product ions of baclofen may be useful in the determination of this compound by less accurate detectors such as a single quadrupole or a triple quadrupole. Depending on the CE, baclofen underwent product ion formation to three major ions at *m*/*z* 197.03638 (CE 5 V), 151.0309 (CE 20 V) and 116.06205 (CE 35 V), which can be used as confirmation ions (Fig. 3).

Based on the MS/MS spectra, the general formula of baclofen fragment ions were suggested (Table 3).

**Table 3:** Accurate mass data obtained by liquid chromatography–quadrupole time-of-flight-mass spectrometry and proposed composition of characteristic product ions of baclofen

No.	Proposed composition	Observed (*m*/*z*)	Calculated (*m*/*z*)	CE (V)	Difference (mDa)	Difference (ppm)	Mass loss	Loss formula
1	C_10_H_9_ClO_2_	197.03383	197.03638	5	2.55	12.96	17.02655	NH_3_
2	C_10_H_10_ClNO	196.05123	196.05237	5	1.14	5.81	18.01056	H_2_O
3	C_10_H_7_ClO	179.02329	179.02582	20	7.86	14.12	35.03711	H_5_NO
4	C_9_H_7_Cl	151.02968	151.0309	20	1.22	8.09	63.03203	CH_5_NO_2_
5	C_7_H_5_Cl	125.01471	125.01525	35	0.54	4.35	89.04768	C_3_H_7_NO_2_
6	C_9_H_7_	116.06136	116.06205	35	0.69	5.96	98.00088	CH_5_ClNO_2_

*CE* collision energy

In its structure, baclofen contains one atom of chlorine, which causes the formation of a characteristic mass spectrum evidencing the presence of isotopic ions of this element [M + H:^35^Cl]^+^ and [M + H:^37^Cl]^+^ (Fig. 3, top panel). The mass difference between chlorine isotopes ^35^Cl and ^37^Cl was 1.9970; therefore, ion fragments with ^35^Cl should be accompanied by the ion containing ^37^Cl, differing by the value mentioned above. Examinations of the MS/MS spectra revealed that several very intense fragments were formed as a result of baclofen fragmentation, depending on the CEs. General formulae were proposed for six of them (Table 3). The use of HRMS enabled to define the precise mass of product ions of interest and errors in their determination as well as to analyze their structure in detail. At a 5-V CE, the most intense fragment was at *m*/*z* 197.03638. The fragment occurred due to dissociation of the ammonium group from the baclofen molecule. Figure 3 also contains the 196.05237 ion, which results from dissociation of a water molecule. This ion was used by Miksa and Poppenga [33] as one of the confirmation ions for determinations of baclofen in bovine serum. In our study, the ion in question was less intense than *m*/*z* 197.03638, which is likely to be associated with a type of ionization different from that applied by Miksa and Poppenga, i.e. atmospheric pressure chemical ionization [33].

**Fig. 4:**
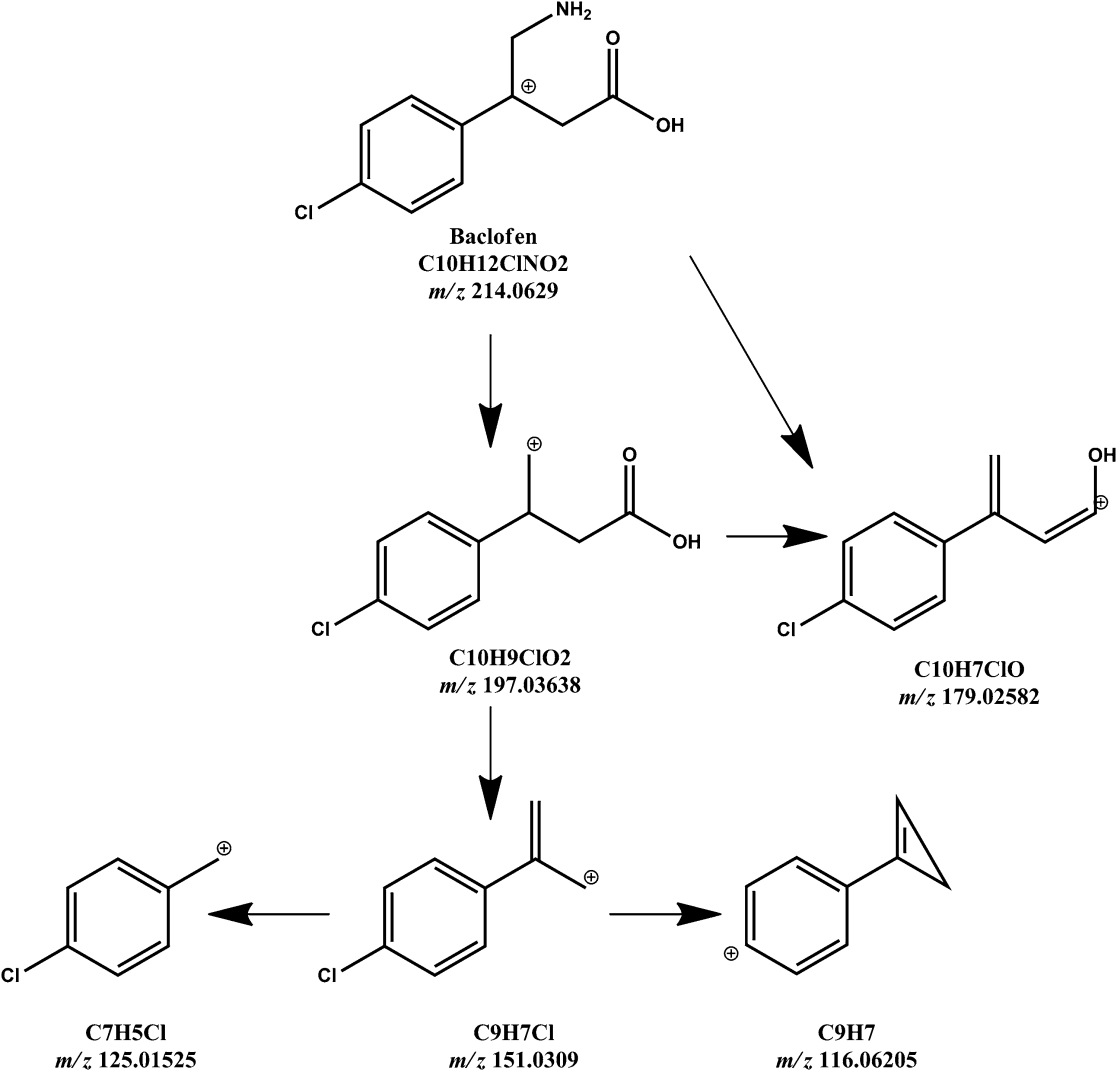
Probable product-ion formation pathways from the protonated baclofen precursor ion

The ion at *m*/*z* 179.02582 (C_10_H_7_^35^ClO) is formed due to dissociation of both the water molecule and ammonium group and not the loss of chlorine (as suggested by Miksa and Poppenga [33]), because the ion at *m*/*z* 181.02282 (C_10_H_7_^37^ClO), characteristic of a chlorine isotope, was also visible in Fig. 3 (top panel). The use of a medium collision energy (CE = 20 V) resulted in the most intense signal coming from the fragment C_9_H_7_Cl at *m*/*z* 151.0309. The signal occurs due to dissociation of the CO_2_ group and ammonium group from the baclofen molecule. The next stages of product formation involve dissociation of the chlorine atom from the ion at *m*/*z* 151.0309 and cyclization of its propylene group, leading to formation of the C_9_H_7_ = 116.06205 fragment, which was the most intense ion at CE = 35 V (Fig. 3, bottom panel and Fig. 4).

In medico-legal toxicology, the cases of fatal intoxication with baclofen alone are rare, as compared to intoxications with other drugs affecting the CNS. The likely causes of baclofen overdose include adaptive and personality disorders as well as depression, leading to suicidal attempts. The literature reports also described cases of accidental consumption of baclofen by children [34–36]. Table 4 lists the cases of baclofen intoxication reported in literature. Only in one case of fatal baclofen intoxication were no other xenobiotics found, which could interact with baclofen. In the case described in the present study, toxicological analysis of the blood demonstrated a concentration of baclofen at 30.7 μg/mL, which markedly exceeds the lethal dose (6–9.6 µg/mL according to TIAFT data) [37]. In only one case described by Giovanni and d’Aloja [18] was the blood concentration of baclofen found to be higher than that in our case; moreover, they identified other xenobiotics in their material as well.

**Table 4:** Literature data regarding cases of baclofen intoxication

No.	Age/sex of victim	Sample/concentration	Method(s)	Other xenobiotic(s)	Outcome	References
1	47/female	Cerebrospinal fluid/0.00338 µg/mL	No data	Not detected	Survived	[38]
2	56/male	Blood/106 µg/mL Urine/774 µg/mL	GC–MS	Dipyrone and metabolites: 4-methyl-aminoantipyrine, oxybutynin	Death	[18]
3	51/female	Blood/2.7 µg/mL	No data	Clorazepate, hydroxyzine, paroxetine, phenobarbital, phenytoin, digoxin, warfarin	Survived	[39]
4	30/female	Urine/no data	No data	Ethanol 312 mg/dL	Survived	[40]
5	47/female	Blood/20 µg/mL	No data	Amitriptyline 1.8 µg/mL, alcohol 160 mg/dL	Death	[41]
6	30/male	Serum/17 µg/mL Urine/760 µg/mL	GC–MS HPLC	Not detected	Death	[19]
7	No data	Blood/0.032 to 2.53 µg/mL	LC–MS/MS	No data	No data	[22]
8	No data	Blood/0.16 to 0.35 µg/mL	LC–MS/MS	No data	No data	[23]
9	43/male	Blood/0.88 µg/mL	LC–MS	No data	Survived	[24]

*GC*–*MS* gas chromatography-mass spectrometry, *HPLC* high-performance liquid chromatography, *LC*–*MS*/*MS* liquid chromatography–tandem mass spectrometry, *LC*–*MS* liquid chromatography–single stage mass spectrometry

## Conclusions

The application of HRMS enables reliable identification of baclofen in autopsy blood. The method designed is characterized by high-efficiency extraction. The study findings demonstrate that the specific, simple and quick procedure described by us for determination of baclofen in autopsy blood can be successfully used for routine toxicology testing in the cases of suspected baclofen intoxication. Unambiguous identification of baclofen is derived from the available product ion mass spectrum. In such a spectrum, we observed three product ions of baclofen (*m*/*z* 197.03638, 151.0309 and 116.06205) of high intensity. These product ions may be successfully employed as confirmative ions in QTOF-MS or triple-quadrupole-MS analysis. Using the obtained tandem mass spectra and defined accurate masses, we proposed the pathways of product ion formation from baclofen. However, the method has a limitation associated with the use of gabapentin as an IS instead of a stable isotope-labeled IS, which is why tested blood should be screened prior to analyzing to ensure that it is free from gabapentin. To our knowledge, this is the first report for analysis of baclofen by HRMS.
